# Biota of coastal wetlands of Praia da Vitória (Terceira Island, Azores): Part 2 - Bryophytes

**DOI:** 10.3897/BDJ.7.e34621

**Published:** 2019-07-01

**Authors:** Rosalina Gabriel, César M.M. Pimentel, David Claro, Mariana R. Brito, Javier Díaz-Castillo, Cecília Sérgio, Manuela Sim-Sim, Paulo Alexandre Vieira Borges

**Affiliations:** 1 CE3C – Centre for Ecology, Evolution and Environmental Changes / Azorean Biodiversity Group and Universidade dos Açores, Angra do Heroísmo, Azores, Portugal CE3C – Centre for Ecology, Evolution and Environmental Changes / Azorean Biodiversity Group and Universidade dos Açores Angra do Heroísmo, Azores Portugal; 2 LIFE CWR – LIFE project “Ecological Restoration and Conservation of Praia da Vitória Coastal Wet Green Infrastructures”, Praia da Vitória, Azores, Portugal LIFE CWR – LIFE project “Ecological Restoration and Conservation of Praia da Vitória Coastal Wet Green Infrastructures” Praia da Vitória, Azores Portugal; 3 CE3C-Centre for Ecology, Evolution and Environmental Changes, Faculdade de Ciências, Universidade de Lisboa, Lisboa, Portugal CE3C-Centre for Ecology, Evolution and Environmental Changes, Faculdade de Ciências, Universidade de Lisboa Lisboa Portugal; 4 Os Montanheiros - Speleological Association, Angra do Heroísmo, Portugal Os Montanheiros - Speleological Association Angra do Heroísmo Portugal

**Keywords:** bryophytes, mosses, liverworts, hornworts, coastal wetlands, bryophyte surveys, standardised sampling, Terceira Island (Azores)

## Abstract

**Background:**

During the LIFE-CWR project "Ecological Restoration and Conservation of *Praia da Vitória* Coastal Wet Green Infrastructures", there was the opportunity to undertake a systematic record of bryophytes at *Paul da Praia da Vitória* (PPV), *Paul do Belo Jardim* (PBJ) and *Paul da Pedreira do Cabo da Praia* (PPCP), three coastal wetland areas of *Praia da Vitória* (Terceira, Azores, Portugal). The objective of the study was to perform a biodiversity assessment, comparing the three sites at two different moments, before and after the implementation of several conservation measures. This project also contributed to improve the knowledge of Azorean bryophyte diversity at both local and regional scales, including the recording of two new taxa for the Azores and three new taxa for Terceira Island.

**New information:**

This paper reports the results of the first extensive survey of bryophyes of the three coastal wetland areas of *Praia da Vitória* (Terceira Island, Azores, Portugal). The identification of a total of 504 samples, 240 collected in 2013 and 2016 (before the LIFE-CWR intervention) and 265 in 2017 and 2018 (after the intervention), resulted in a list of 58 species of bryophytes (one hornwort, eight liverworts and 48 mosses). These include two new records for the Azores (*Bryum klinggraeffii*, *Ptychostomum bornholmense*), three new records for Terceira Island (*Bryum tenuisetum*, *Dicranella howei*, *Trichostomum crispulum*) and at least 15 new records for the municipality of Praia da Vitória (e.g., *Cephaloziella hampeana*). Some species that had not been recorded on the island since 1937 (e.g., *Fissidens crispus*) were collected during this study, as well as a Macaronesian endemic liverwort (*Radula wichurae*), an Iberian-Macaronesian liverwort (*Frullania azorica*) and a moss species with European distribution (*Ptychomitrium nigrescens*). From the recorded species, only one moss (*Leptophascum leptophyllum*), of subtropical origin, is considered invasive in Europe.

## Introduction

Bryophytes are small plants without vascular tissues, directly depending on immediate environmental conditions. Most species are poikilohydric, i.e., lacking the ability (structural or functional) to maintain and/or regulate water content to achieve homeostasis of cells and tissue. Because they are so dependant on their immediate environment, they respond quickly to environmental change, which makes them good bioindicators of changes in land use, precipitation regime, temperature, salinity and pollution.

The Azores archipelago is well-known for its rich bryoflora (480 species and subspecies) (Gabriel et al. 2010), which may be related to the high humidity and mild temperatures, influenced by the Atlantic Ocean, and scarce pollution sources.

Although the coastal areas of the islands are among their most deteriorated habitats, mainly due to urbanization pressure, some interesting, though fragmented, ecosystems remain at lower elevations in the Azores. The three coastal wetlands of *Praia da Vitória* municipality are a case in point, well worth restoration and habitat protection. The areas, studied during the LIFE-CWR Project – *Paul da Praia da Vitória* (PPV), *Paul do Belo Jardim* (PBJ) and *Paul da Pedreira do Cabo da Praia* (PPCP) – are best known for their birds (Barcelos et al. 2015; Dias 2018; Goulart et al. 2019); however, they harbour other important and interesting biological groups, such as molluscs and arthropods (Martins and Borges 2019), plants and lichens (Elias et al. 2019).

The coastal areas of the Azorean Islands are not thoroughly studied, since bryologists tend to focus on the rich natural forests of the archipelago (e.g., Aranda et al. 2011) and the bryophytic flora of these coastal wetlands had never been systematically sampled.

This is the second contribution in a series of papers (Borges et al. 2018) intending to characterize the biota of the three areas.

## General description

### Purpose

The main aim of this work was to inventory the bryophyte species present in *Paul da Praia da Vitória* (PPV), *Paul do Belo Jardim* (PBJ) and *Paul da Pedreira do Cabo da Praia* (PPCP), three neighbouring areas focused on by the restoration project LIFE-CWR, in order to improve knowledge on the regional distribution of bryophytes (mosses, liverworts, hornworts) and set a baseline for future research in the area.

## Project description

### Title

Inventory of bryophytes in three coastal wetlands of Terceira Island (Azores)

### Personnel

The inventory was conducted during the years of 2013, 2016, 2017 and 2018 under the responsibility of Rosalina Gabriel, with the participation of Javier Diaz Castillo (2013), César Pimentel and Mariana Reis Brito (2016, 2017). Sampling dates and collectors are listed in Table 1. Species identifications were performed by César Pimentel under the supervision of Rosalina Gabriel (2016 and 2017), and David Claro (2013) under the supervision of Cecília Sérgio. The identification of some challenging samples was performed by Manuela Sim-Sim and Cecília Sérgio.

### Study area description

Terceira Island (area: 400.6 km²; elevation: 1,021.14 m) is one of the nine islands of the Azores archipelago, located in the North Atlantic, roughly at 38°43′49″N, 27°19′10″W (Forjaz 2004). The climate in the Azores is temperate oceanic, with regular and abundant rainfall, high levels of relative humidity and persistent western winds, mainly during the winter and autumn seasons (Azevedo et al. 2004).

**Figure 1.**
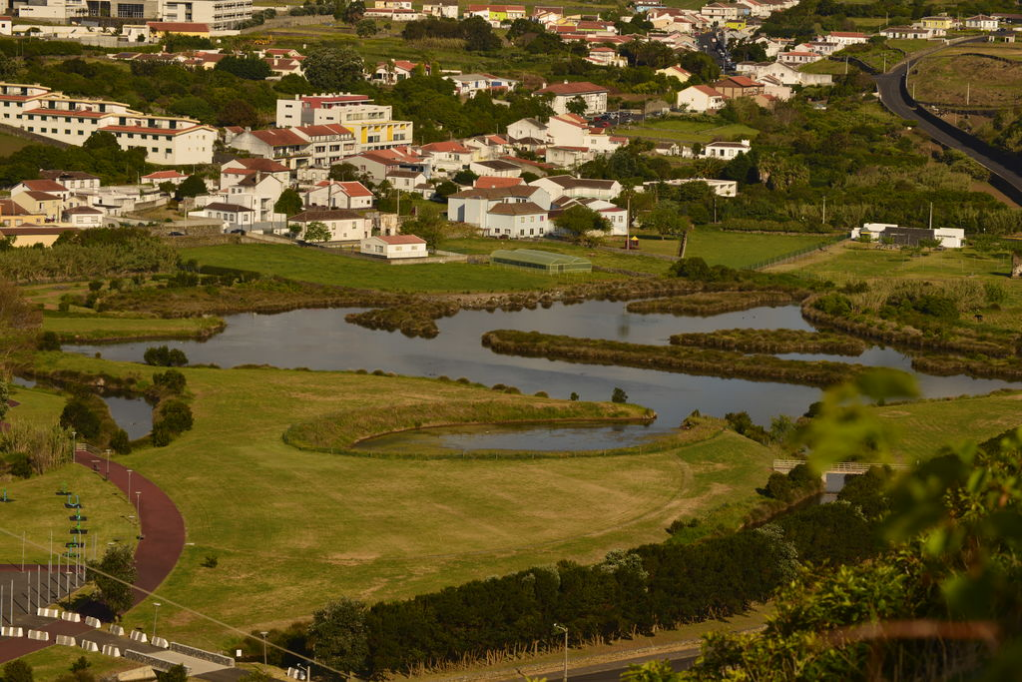
General aspect of *Paul da Praia da Vitória* with its islands and surrounding urban area (Photo by Paulo A.V. Borges).

**Figure 2.**
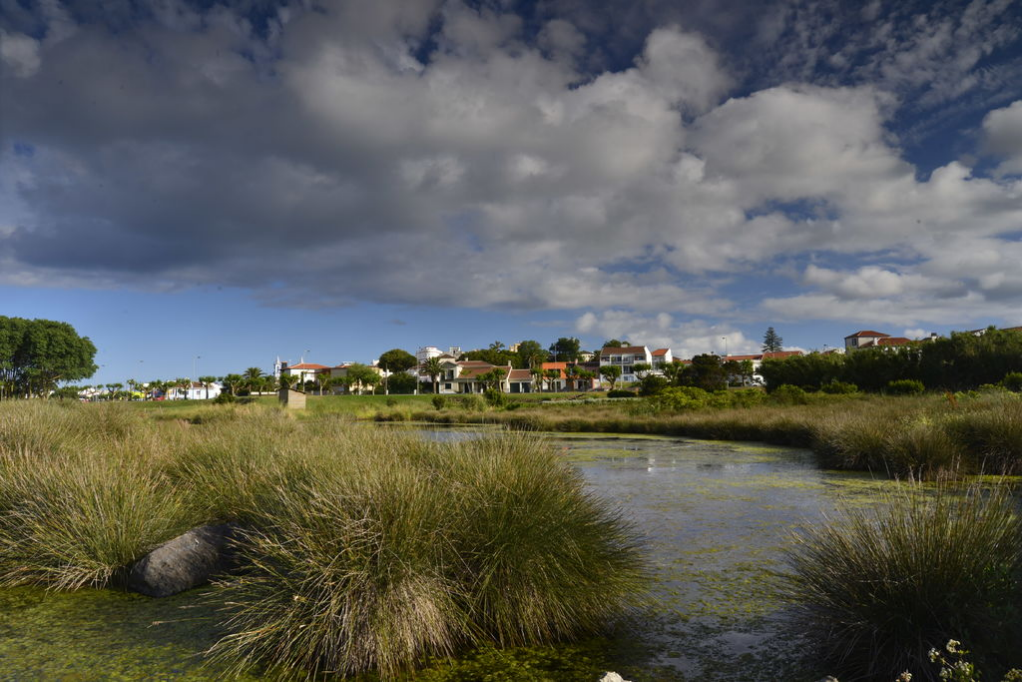
Detail of the recently created "islands" of *Juncus acutus* in *Paul da Praia da Vitória* (Photo by Paulo A.V. Borges).

**Figure 3.**
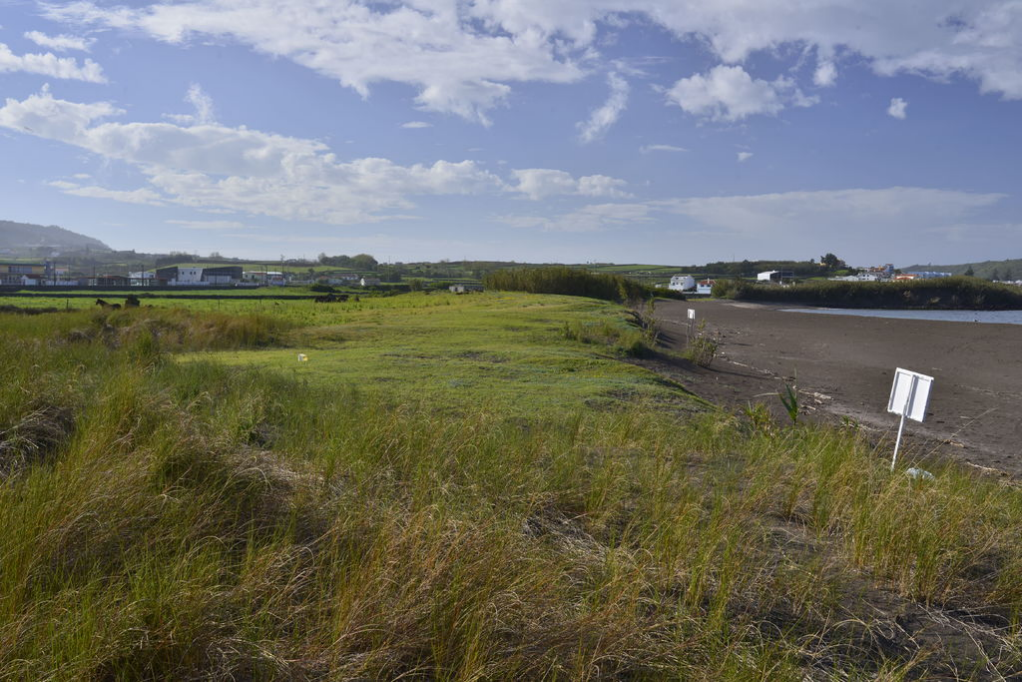
*Paul do Belo Jardim*' s dune area (Photo by Paulo A.V. Borges).

**Figure 4.**
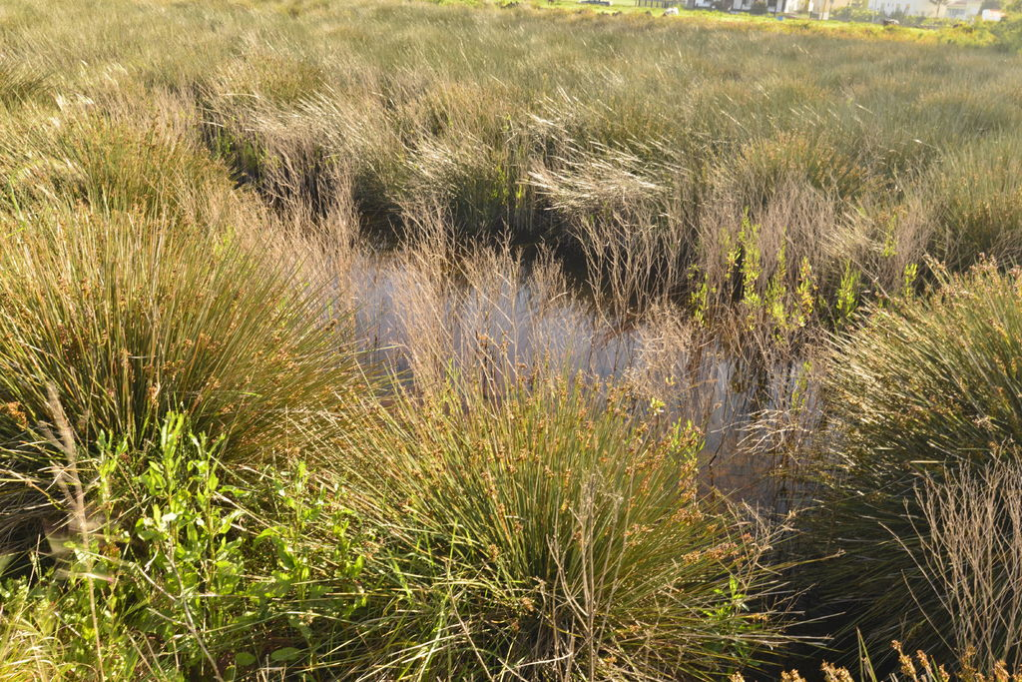
*Juncus maritimus* growing in the *Paul do Belo Jardim* area (Photo by Paulo A.V. Borges).

**Figure 5.**
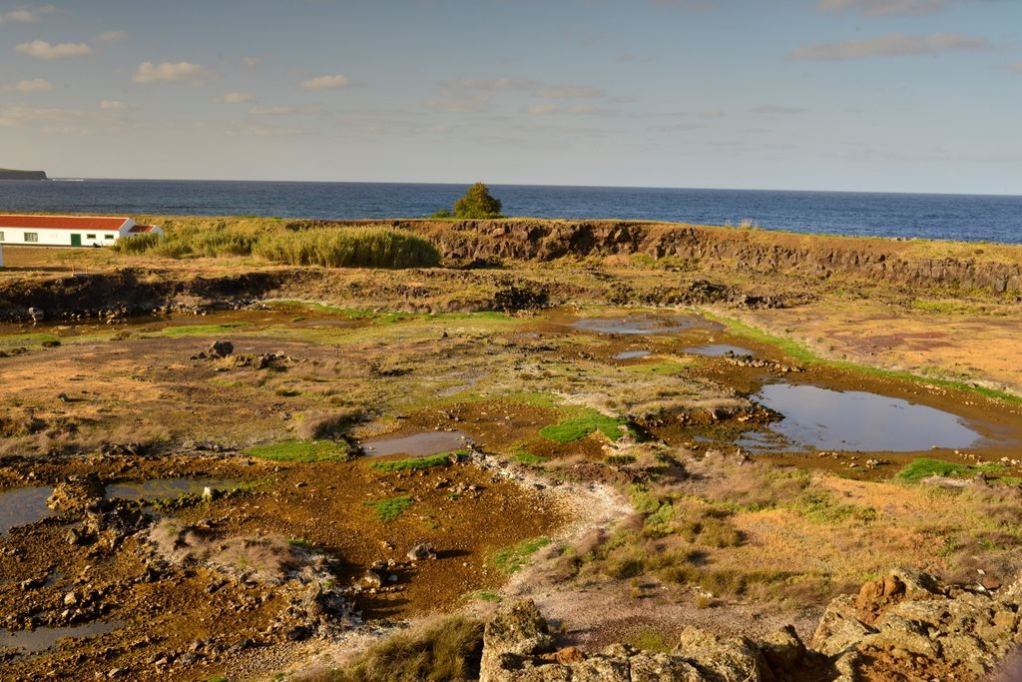
General view of *Paul da Pedreira do Cabo da Praia*, at low tide (Photo by Paulo A.V. Borges).

**Figure 6.**
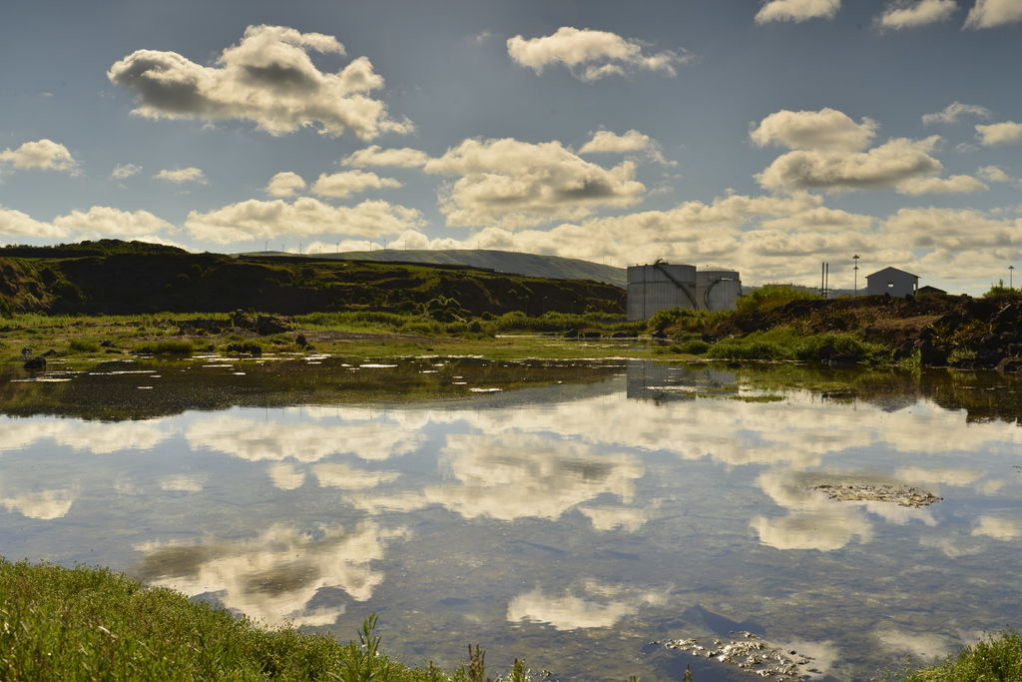
Detail of the margin of *Paul da Pedreira do Cabo da Praia* during high tide (Photo by Paulo A.V. Borges).

Terceira Island is known for the presence of some very important native forest areas at high elevation (e.g., Gabriel and Bates 2005). However, few natural areas remain at lower elevations, notably in Praia da Vitória county. Three coastal wetland areas were studied in this project: *Paul da Praia da Vitória* (PPV) (Figs 1, 2), *Paul do Belo Jardim* (PBJ) (Figs 3, 4) and *Paul da Pedreira do Cabo da Praia* (PPCP) (Figs 5, 6).

Potentially, the plant cover of Praia da Vitória would include *Erica-Morella* coastal woodlands (cf. Elias et al. 2016). However, apart from some native shrubs of *Morella faya*, still found in PPV and PPCP, the main native species found in the area include species typical of humid zones, namely *Juncus* (*J. acutus*, *J. maritimus*, *J. effusus*) and *Ruppia maritima* (Elias et al. 2019). Presently, most of the area is covered with exotic and invasive species, with the giant cane (*Arundo donax*), being especially abundant in PBJ, and the sticky snakeroot (*Ageratina adenophora*) in PPCP. Both these species are included in the first quartile of invasive species in the three archipelagos of Macaronesia (Silva et al. 2008). The bryophytic component of the flora had previously not been systematically studied in the area.

### Design description

In each wetland, a network of three (PBJ) or four (PPV, PPCP) transects (160 m × 2 m or 300 m × 2 m), was set and sampled every 20 m (ocasionally every 10 m), in quadrats/sampling points of 4 m^2^; each quadrat was searched for bryophytes. Whenever possible, a maximum of six samples or microplots (10 cm × 5 cm) were collected: three replicates from soil and three replicates from rock. Bryophyte samples were brought to the laboratory for identification and herborization at the Herbarium of the University of Azores (AZU) – section Bryophytes.

In 2018, after the project's completion, the areas continued to be visited and in one of those visits a new species for the *Paul da Pedreira do Cabo da Praia* location was collected.

### Funding

This study was financed by the project LIFE+ (LIFE12 BIO/PT/000110: Ecological Restoration and Conservation Infrastructure Green Wet Coast Praia da Vitória) (2013–2018).

## Sampling methods

### Study extent

This study coverered a small coastal area, extending from PPV (to the North) to PPCP (to the South), with an extent of 3.58 km.

### Sampling description

In each site, bryophytes were sampled using standardised methods, during one or two visits in 2013, 2016 and 2017, respectively (Table 1). Within each transect, a quadrat with an area of 4 m^2^ was delimited at intervals of 10 or 20 meters. Each sampling point (quadrat) was carefully examined to collect three samples (replicates or microplots) for each of the available substrates (soil, rock); microplots were randomly selected from areas colonized by bryophytes. The area collected in each replica (microplot) was 50 cm^2^ (10 cm × 5 cm). The maximum number of samples obtained per sampling point was six (three replicates of bryophytes growing on soil and three of bryophytes growing on rock), but many sampling points did not contain bryophytes, especially those located in areas periodically flooded with brackish waters. For each microplot, some ecological variables were also measured (e.g., insulation, water availability) using ordinal scales adapted from Gabriel and Bates (2005), and some soil or rock was recovered for pH measurement.

### Quality control

The correct identification of the sampled taxa is crucial in an inventory. Keys and floras were used to identify the species, and their coverage (in %) was also estimated for each microplot in the laboratory. The main floras used for the identification of liverworts were by Paton (1999) and Casas et al. (2009), whereas for mosses Smith (2004), Casas et al. (2006) and different volumes of "Flora Briofítica Ibérica" (Guerra 2018) were used. Taxonomic keys provided by Schumacker and Váňa (2005) and field guides (Atherton et al. 2010, Llimona et al. 2004) were also checked. Some important internet databases were consulted, namely the Azorean Biodiversity Portal and TROPICOS for taxonomic data and BBS Field Guide online pages, Bildatlas der Moose Deutschlands and Swissbryophytes for morphological and ecological data. Nomenclature mostly follows Gabriel et al. (2010) and Ros et al. 2013.

Samples were mostly examined by CP and DC, and their identification was supervised by RG and CS.

## Geographic coverage

### Description

Praia da Vitória municipality, Terceira Island, Azores archipelago, Macaronesia, Portugal.

### Coordinates

38º42'09''N and 38°44'12''N Latitude; 27º03'46''W and 27°02'39''W Longitude.

## Taxonomic coverage

### Description

Bryophytes, including Division Anthocerotophyta, Division Bryophyta, and Division Marchantiophyta.

## Temporal coverage

### Notes

The main sampling was performed in 2013, 2016 and 2017; a single sample was collected in 2018.

## Usage rights

### Use license

Open Data Commons Attribution License

### IP rights notes

Additional information on this study may also be requested from the first author.

## Data resources

### Data package title

LIFE_CWR_TER_Bryophytes

### Resource link

http://ipt.gbif.pt/ipt/resource?r=bryophytes_vitoria_azores

### Alternative identifiers

http://islandlab.uac.pt/software/ver.php?id=31

### Number of data sets

1

### Data set 1.

#### Data set name

Bryophytes from Praia da Vitória.

#### Data format

Darwin Core Archive.

#### Number of columns

56

#### Download URL

http://ipt.gbif.pt/ipt/resource?r=bryophytes_vitoria_azores

#### Data format version

1.

#### Description

In this data table, we include all records for which a taxonomic identification of the species was possible. The dataset submitted to GBIF is structured as a sample event dataset, with two tables: event (as core) and occurrences. The data in this sampling event resource have been published as a Darwin Core Archive (DwC-A), which is a standardised format for sharing biodiversity data as a set of one or more data tables. The core data table contains 188 events. One extension data table also exists. An extension record supplies extra information about a core record. The number of records in each extension data table is illustrated in the IPT link.

This IPT archives the data and thus serves as the data repository. The data and resource metadata are available for downloadin the downloads section. The versions table lists other versions of the resource that have been made publicly available and allows tracking changes made to the resource over time.

**Data set 1.** 

Column label	Column description
Table Event	The sub-table with events
id	Identifier of the events, unique for the dataset
type	Type of the record, as defined by the Public Core standard
license	Reference to the license under which the record is published
institutionID	The identity of the institution publishing the data
institutionCode	The code of the institution publishing the data
datasetName	Name of the dataset
eventID	Identifier of the events, unique for the dataset
samplingProtocol	The sampling protocol used to capture the species
samplingEffort	The amount of time of each sampling
eventDate	The date-time or interval during which an Event occurred. For occurrences, this is the date-time when the event was recorded.
startDayOfYear	The earliest ordinal day of the year on which the Event occurred
habitat	The habitat for an Event
continent	The name of the continent in which the Location occurs
islandGroup	The name of the island group in which the Location occurs
island	The name of the island on or near which the Location occurs
country	The name of the country or major administrative unit in which the Location occurs
countryCode	The standard code for the country in which the Location occurs
municipality	The full, unabbreviated name of the next smaller administrative region than county (city, municipality, etc.) in which the Location occurs
locality	The specific description of the place
verbatimCoordinates	Original coordinates recorded
decimalLatitude	Approximate centre point decimal latitude of the field site in GPS coordinates
decimalLongitude	Approximate centre point decimal longitude of the field site in GPS coordinates
Table Occurrences	The sub-table with occurrence data
id	Identifier of the events, unique for the dataset
license	Reference to the license under which the record is published
institutionID	The identity of the institution publishing the data
institutionCode	The code of the institution publishing the data
collectionCode	The code of the collection where the specimens are conserved
datasetName	Name of the dataset
basisOfRecord	The nature of the data record
dynamicProperties	A list of additional measurements, facts, characteristics, or assertions about the record. Meant to provide a mechanism for structured content
occurrenceID	Identifier of the record, coded as a global unique identifier
occurrenceRemarks	Remarks on the occurrence substracte from where the specimens were captured
recordNumber	An identifier given to the Occurrence at the time it was recorded
recordedBy	A list (concatenated and separated) of names of people, groups, or organizations responsible for recording the original Occurrence
organismQuantity	A number or enumeration value for the quantity of organisms
organismQuantityType	The unit of the identification of the organisms
establishmentMeans	The process of establishment of the species in the location, using a controlled vocabulary: 'native non-endemic', 'introduced', 'endemic'
disposition	The current state of a specimen with respect to the collection identified in collectionCode or collectionID
eventID	Identifier of the events, unique for the dataset
fieldNumber	An identifier given to the event in the field
minimumElevationInMeters	Minimum elevation in metres
identifiedBy	Name of the person who made the identification
dateIdentified	Date on which the record was identified
scientificName	Complete scientific name including author
kingdom	Kingdom name
phylum	Phylum name
class	Class name
order	Order name
family	Family name
genus	Genus name
specificEpithet	Specific epithet
infraspecificEpithet	Infraspecific epithet
taxonRank	Lowest taxonomic rank of the record
scientificNameAuthorship	The authorship information for the scientificName formatted according to the conventions of the applicable nomenclaturalCode

## Additional information

The identification of the samples (242 before the LIFE-CWR intervention [2013, 2016], 261 after it [2017]) resulted in a set of 57 species of bryophytes, including one hornwort, eight liverwort species (Table 2) and 48 moss species (Table 3), representing about 80% of the bryophyte species present in the three sampled areas, according to the first-order Jackknife estimator (Table 4) (Gabriel 2018).

**Comparison between years (before and after CWR intervention)**: The main interventions performed by the LIFE-CWR project in the three coastal areas included the removal of garbage and litter from PPCP, the opening of a small lagoon in PBJ and the connection of PPV to the sea.

The number of species varied slightly before and after the interventions, but the level of completeness is acceptable, higher than 75%, for both sampling periods (Table 4). The highest value of bryophyte species richness was observed in Paul da Pedreira do Cabo da Praia, probably due to the availability of a higher proportion of rocky substrata, while the lowest richness value was observed in Paul da Praia da Vitória.

**Figure 7.**
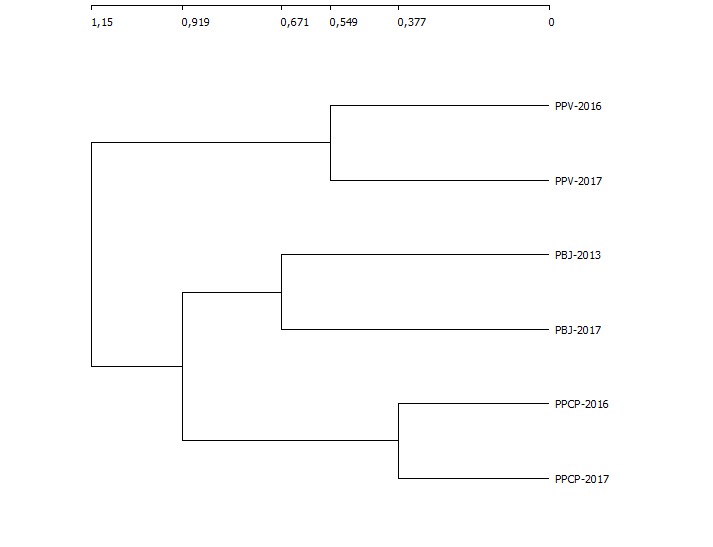
Dendogram showing the result of a Ward's dissimilarity analysis including the bryophytes from the three wetlands of the municipality of Praia da Vitória (Terceira Island, Azores) before (2013, 2016) and after (2017) the interventions made by the LIFE-CWR project.

A Ward's dissimilarity analysis performed with the diversity of species found at the three studied wetlands shows a remarkable homogeinity of results between the studied years (Fig. 7). Thus, the LIFE-CWR restoration interventions, especially focused on the improvement the bird habitat and water flow, did not hinder the conservation of bryophytes.

**Main biogeographic distribution of the species**: Most species found in the three studied wetlands have a broad biogeographic distribution, generally circumpolar and European, showing temperate climatic characteristics. Although most of the collected species are common in the Azores, three species are classified as Rare by IUCN (*Grimmia lisae, Tortula solmsii* and *Riccia huebeneriana*) (Dierssen 2001), and a single moss species (*Leptophascum leptophyllum*), of subtropical origin, is considered invasive in Europe. This species is widespread in humanized areas and may commonly be found on the sidewalks of some cities (Blockeel et al. 2014).

**Noteworthy species**: Among the observed species, two represent new records for the Azores, *Bryum klinggraeffii* (Ellis et al. 2016) and *Ptychostomum bornholmense* (Ellis et al. 2018). There are also three new records for Terceira Island (*Bryum tenuisetum, Dicranella howei* and *Trichostomum crispulum*) and at least 15 new recordsfor the municipality of Praia da Vitória, including the moss *Leucodon sciuroides*, a species previously known only from Monte Brasil (Angra do Heroísmo, Terceira Island) (Fontinha and Sérgio 1995) and which has been declining in the United Kingdom (Blockeel et al. 2014), and the liverwort *Cephaloziella hampeana*, also known from a single location on Terceira Island (Algar do Carvão) (Crundwell et al. 2013).

Some species that had not been recorded on Terceira Island since 1937 (e.g., *Fissidens crispus*) (Gabriel et al. 2011) were found on the wetlands, which may be explained by a lack of fieldwork at low elevations on the island (Aranda et al. 2011).

**Figure 8.**
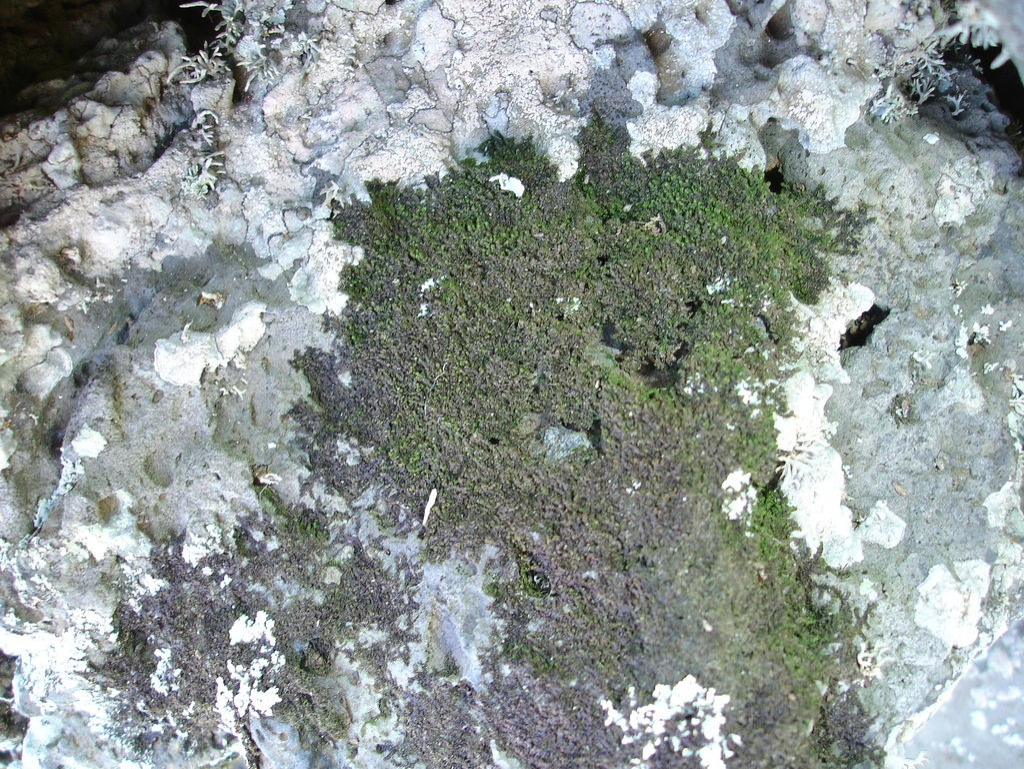
The liverwort *Frullania azorica* was described from material collected in the Praia da Vitória area (Photo by Rosalina Gabriel).

A Macaronesian endemic liverwort (*Radula wichurae*) and an Iberian-Macaronesian liverwort (*Frullania azorica*; Fig. 8) were found growing on rocks in the different wetlands. Actually, Praia da Vitória county, parish of Cabo da Praia, represents the classical locality of *Frullania azorica*, the place from where the species was originally collected and described (Sim-Sim et al. 1995). This species is frequently found in the area, sometimes forming extensive colonies on exposed rocks near the ocean. However, in this study, it was not identified in Paul da Praia da Vitória, possibly because there are not many rocks available for colonization.

The acrocarpic moss species *Ptychomitrium nigrescens*, endemic to Europe and Macaronesia (Macaronesia, Portugal and France), was also reported from *Paul da Pedreira do Cabo da Praia* (PPCP), where boulders and large rocks are available for colonization.

Further details related to the LIFE-CWR project can be found in the book by Brian Morton, Elisabete Nogueira and António Frias Martins (Morton et al. 2019) and in the report by RG (Gabriel 2018).

## Figures and Tables

**Table 1.** Dates of collection and collectors of bryophytes from the three wetlands of the county of Praia da Vitória (PPV - Paul da Praia da Vitória; PBJ - Paul do Belo Jardim; PPCP - Paul da Pedreira do Cabo da Praia).

**Area**	**Year**	**Sampling date**	**Transects**	**Latitude**	**Longitude**	**Collectors**
PPV	2016	31-03-2016	1	38,73534	-27,06042	César Pimentel
		01-04-2016	2	38,73449	-27,05833	César Pimentel & Mariana R. Brito
		28-06-2016	3	38,73590	-27,06027	César Pimentel & Mariana R. Brito
		28-06-2016	4	38,73443	-27,05944	César Pimentel & Mariana R. Brito
	2017	12-06-2017	1A	38,73443	-27,05944	Rosalina Gabriel, César Pimentel & Mariana R. Brito
			2A	38,73534	-27,06042	Rosalina Gabriel, César Pimentel & Mariana R. Brito
			3A	38,73449	-27,05833	Rosalina Gabriel, César Pimentel & Mariana R. Brito
			4A	38,73590	-27,06027	Rosalina Gabriel, César Pimentel & Mariana R. Brito
PBJ	2013	18-10-2013	1A	38,71337	-27,06080	Javier Diaz Castillo
		28-10-2013	1B	38,71298	-27,06102	Javier Diaz Castillo
			2A	38,71389	-27,06119	Javier Diaz Castillo
		01-11-2013	2B	38,71317	-27,06123	Javier Diaz Castillo
	2017	13-06-2017	1	38,71355	-27,06182	Rosalina Gabriel, César Pimentel & Mariana R. Brito
			2	38,71350	-27,06107	Rosalina Gabriel, César Pimentel & Mariana R. Brito
			3	38,71333	-27,06113	Rosalina Gabriel, César Pimentel & Mariana R. Brito
PPCP	2016	24-02-2016	1	38,70367	-27,04556	Rosalina Gabriel, César Pimentel & Mariana R. Brito
			2	38,70327	-27,04553	Rosalina Gabriel, César Pimentel & Mariana R. Brito
		13-05-2016	3	38,70440	-27,04513	Rosalina Gabriel, César Pimentel & Mariana R. Brito
			4	38,70267	-27,04801	Rosalina Gabriel, César Pimentel & Mariana R. Brito
	2017	14-06-2017	1A	38,70367	-27,04556	Rosalina Gabriel, César Pimentel & Mariana R. Brito
			2A	38,70327	-27,04553	Rosalina Gabriel, César Pimentel & Mariana R. Brito
			3A	38,70440	-27,04513	Rosalina Gabriel, César Pimentel & Mariana R. Brito
			4A	38,70267	-27,04801	Rosalina Gabriel, César Pimentel & Mariana R. Brito
	2018	01-09-2018	na	38,70306	-27,04745	Rosalina Gabriel & Paulo A.V. Borges

**Table 2.** List of hornworts (Division Anthocerotophyta) and liverworts (Division Marchantiophyta) identified in the three coastal wetlands of Praia da Vitória, Terceira Island, Azores, Portugal (PPV – Paul da Praia da Vitória; PBJ – Paul do Belo Jardim; PPCP – Paul da Pedreira do Cabo da Praia), indicating their Class, Order and Family as well as the number of samples obtained for each species during LIFE-CWR fieldwork.

**Class**	**Order**	**Family**	**Species/Subspecies**	**PPV**	**PBJ**	**PPCP**
Anthocerotopsida	Notothyladales	Notothyladaceae	*Phaeoceros laevis* (L.) Prosk.	1	2	
Jungermanniopsida	Fossombroniales	Fossombroniaceae	Fossombronia caespitiformis De Not. ex Rabenh. subsp. multispira (Schiffn.) J. R. Bray et D. C. Cargill			9
Jungermanniopsida	Jungermanniales	Cephaloziellaceae	*Cephaloziella hampeana* (Nees) Schiffn.			2
Jungermanniopsida	Porellales	Frullaniaceae	*Frullania azorica* Sim-Sim et al.		2	8
Jungermanniopsida	Porellales	Lejeuneaceae	*Marchesinia mackaii* (Hook.) Gray			1
Jungermanniopsida	Porellales	Radulaceae	*Radula lindenbergiana* Gottsche ex C. Hartman			1
Jungermanniopsida	Porellales	Radulaceae	*Radula wichurae* Steph.			2
Marchantiopsida	Lunulariales	Lunulariaceae	*Lunularia cruciata* (L.) Dumort ex. Lindb.	1	8	7
Marchantiopsida	Marchantiales	Ricciaceae	*Riccia huebeneriana* Lindenb.			1

**Table 3.** List of mosses (Division Bryophyta) identified in the three coastal wetlands of Praia da Vitória, Terceira Island, Azores, Portugal (PPV – Paul da Praia da Vitória; PBJ – Paul do Belo Jardim; PPCP – Paul da Pedreira do Cabo da Praia), indicating their Class, Order and Family as well as the number of samples obtained for each species during LIFE-CWR fieldwork.

**Class**	**Order**	**Family**	**Species/Subspecies**	**PPV**	**PBJ**	**PPCP**
Bryopsida	Bartramiales	Bartramiaceae	*Philonotis marchica* (Hedw.) Brid.			7
Bryopsida	Bartramiales	Bartramiaceae	*Philonotis rigida* Brid.	1		2
Bryopsida	Bryales	Bryaceae	*Anomobryum julaceum* (P. Gaerth., B. Mey. et Scherb.) Schimp.			45
Bryopsida	Bryales	Bryaceae	*Bryum argenteum* Hedw.		9	
Bryopsida	Bryales	Bryaceae	*Bryum canariense* Brid.			4
Bryopsida	Bryales	Bryaceae	*Bryum klinggraeffii* Schimp.		4	
Bryopsida	Bryales	Bryaceae	*Bryum ruderale* Crundw. et Nyholm	5	1	
Bryopsida	Bryales	Bryaceae	*Bryum subapiculatum* Hampe	1	14	
Bryopsida	Bryales	Bryaceae	*Bryum tenuisetum* Limpr.	1		16
Bryopsida	Bryales	Bryaceae	*Ptychostomum capillare* (Hedw.) D. T. Holyoak et N. Pedersen		36	84
Bryopsida	Bryales	Bryaceae	*Ptychostomum dichotomum* Hedw.		1	
Bryopsida	Bryales	Bryaceae	*Ptychostomum bornholmense* (Wink. & R.Ruthe) Holyoak & N.Pedersen		7	
Bryopsida	Bryales	Bryaceae	*Ptychostomum pseudotriquetrum* (Hedw.) J. R. Spence et H. P. Ramsay ex D. T. Holyoak et N. Pedersen			1
Bryopsida	Bryales	Bryaceae	*Ptychostomum rubens* (Mitt.) D. T. Holyoak et N. Pedersen	12	6	2
Bryopsida	Dicranales	Dicranaceae	*Dicranella howei* Renauld et Cardot		1	1
Bryopsida	Dicranales	Ditrichaceae	Ceratodon purpureus (Hedw.) Brid. subsp. purpureus			4
Bryopsida	Dicranales	Fissidentaceae	*Fissidens crispus* Mont.	61	7	12
Bryopsida	Dicranales	Fissidentaceae	*Fissidens viridulus* (Sw. ex anon.) Wahlenb.			1
Bryopsida	Dicranales	Leucobryaceae	*Campylopus pilifer* Brid.			31
Bryopsida	Grimmiales	Grimmiaceae	*Grimmia lisae* De Not.	3	11	57
Bryopsida	Grimmiales	Ptychomitriaceae	*Ptychomitrium nigrescens* (Kunze) Wijk et Marg.			1
Bryopsida	Hypnales	Brachytheciaceae	*Brachytheciastrum velutinum* (Hedw.) Ignatov et Huttunen	7	1	4
Bryopsida	Hypnales	Brachytheciaceae	*Brachythecium mildeanum* (Schimp.) Milde		1	
Bryopsida	Hypnales	Brachytheciaceae	*Brachythecium rutabulum* (Hedw.) Schimp.	5	1	12
Bryopsida	Hypnales	Brachytheciaceae	*Brachythecium plumosum* (Hedw.) Schimp.		1	
Bryopsida	Hypnales	Brachytheciaceae	*Kindbergia praelonga* (Hedw.) Ochyra	7	3	10
Bryopsida	Hypnales	Brachytheciaceae	*Oxyrrhynchium hians* (Hedw.) Loeske	1		1
Bryopsida	Hypnales	Brachytheciaceae	*Oxyrrhynchium speciosum* (Brid.) Warnst.	1		
Bryopsida	Hypnales	Brachytheciaceae	*Rhynchostegiella litorea* (De Not.) Limpr.	3		
Bryopsida	Hypnales	Brachytheciaceae	*Rhynchostegium confertum* (Dicks.) Schimp.	17	6	19
Bryopsida	Hypnales	Brachytheciaceae	*Rhynchostegium megapolitanum* (F. Weber et D. Mohr.) Schimp.			1
Bryopsida	Hypnales	Hypnaceae	Hypnum cupressiforme Hedw. var. cupressiforme			15
Bryopsida	Hypnales	Leucodontaceae	*Leucodon sciuroides* (Hedw.) Schwägr.			1
Bryopsida	Pottiales	Pottiaceae	*Barbula convoluta* Hedw.	1	8	12
Bryopsida	Pottiales	Pottiaceae	*Barbula unguiculata* Hedw.	1		
Bryopsida	Pottiales	Pottiaceae	*Didymodon australasiae* (Hook. & Grev.) R.H. Zander			6
Bryopsida	Pottiales	Pottiaceae	*Didymodon sicculus* M.J. Cano, Ros, García-Zam. & J. Guerra			11
Bryopsida	Pottiales	Pottiaceae	*Didymodon tophaceus* (Brid.) Lisa	1		
Bryopsida	Pottiales	Pottiaceae	*Didymodon umbrosus* (Müll. Hal.) R.H. Zander	5	3	10
Bryopsida	Pottiales	Pottiaceae	*Leptophascum leptophyllum* (Müll. Hal.) J. Guerra et M. J. Cano		8	1
Bryopsida	Pottiales	Pottiaceae	*Pseudocrossidium hornschuchianum* (Schultz) R. H. Zander		1	
Bryopsida	Pottiales	Pottiaceae	*Tortella flavovirens* (Bruch.) Broth.	3	10	23
Bryopsida	Pottiales	Pottiaceae	*Tortula muralis* Hedw.	3	3	2
Bryopsida	Pottiales	Pottiaceae	*Tortula solmsii* (Schimp.) Limpr.	2		1
Bryopsida	Pottiales	Pottiaceae	*Tortula truncata* (Hedw.) Mitt.		1	
Bryopsida	Pottiales	Pottiaceae	*Trichostomum brachydontium* Bruch	14	12	119
Bryopsida	Pottiales	Pottiaceae	*Trichostomum crispulum* Bruch		2	15
Bryopsida	Pottiales	Pottiaceae	*Weissia controversa* Hedw.			3
Polytrichopsida	Polytrichales	Polytrichaceae	*Polytrichum piliferum* Hedw.			1

**Table 4.** Some statistical data from the collection of bryophytes in Praia da Vitória wetlands per year of collection (Number of samples; Observed richness or number of species (S); Number of estimated richness according to the first order Jackknife estimator; Percentage of completeness, i.e., ratio between the number of estimated species and the number of observed of species).

		**2013|2016**	**2017**
**PPV**	Number of samples	44	83
	Observed richness (S)	14	17
	Estimated richness	17,91	22,93
	% **Completeness**	**78,17**	**74,14**
**PBJ**	Number of samples	42	58
	Observed richness (S)	14	24
	Estimated richness	18,88	32,84
	% **Completeness**	**74,15**	**73,08**
**PPCP**	Number of samples	156	120
	Observed richness (S)	33	28
	Estimated richness	43,93	32,96
	% **Completeness**	**75,12**	**84,95**
**TOTAL**	Number of samples	242	261
	Observed richness (S)	45	42
	Estimated richness	57,95	52,96
	% **Completeness**	**77,65**	**79,31**
